# Religiosity Among Young-Adult Baby Boomers: Associations With Psychological Well-Being Over 45 Years

**DOI:** 10.1093/geroni/igab046.1954

**Published:** 2021-12-17

**Authors:** Kent Jason Cheng, Maria Brown, Woosang Hwang, Merril Silverstein

**Affiliations:** 1 Syracuse University, Syracuse, New York, United States; 2 Syracuse University, Syracuse, New York, New York, United States

## Abstract

Past studies on the influence of religiosity on psychological well-being tended to be cross-sectional in nature and neglected generational differences. In this study, we assess how religiosity in early adulthood (mean age = 19) affects baby-boomers’ psychological well-being over the life course. We used waves 1 to 9 or 45 years of survey data from the Longitudinal Study of Generations (LSOG) (N=798), a sample of Southern Californians. First, we used latent class analyses on five domains to identify three typologies of baby boomers’ religiosity in early adulthood. We call these typologies “strongly religious,” “weakly religious,” and “personally religious.” Then, we used latent growth curve modelling to ascertain the influence of these religiosity typologies on psychological wellbeing from waves 1 to 9, controlling for time-invariant (religious affiliation, age, sex, race, parental income) and varying (religious salience, education, marital status, and annual income) factors. We found that the strongly religious have a consistently upward psychological wellbeing trend throughout the study period whereas wellbeing started to decline for the weakly religious and personally religious at around wave 6, on when they were about mid-40s to almost 50. We provide evidence that religiosity in early adulthood – a period in life characterized by the exploration of various options for the future brought about by greater personal freedom – positively influences baby boomer’s psychological wellbeing over the life course.

